# The socio-demographic, clinical characteristics and outcomes of tuberculosis among HIV infected adults in Lithuania: A thirteen-year analysis

**DOI:** 10.1371/journal.pone.0282046

**Published:** 2023-03-23

**Authors:** Elzbieta Matulyte, Edita Davidaviciene, Zavinta Kancauskiene, Saulius Diktanas, Aidas Kausas, Daiva Velyvyte, Jurgita Urboniene, Vilnele Lipnickiene, Megan Laurencikaite, Edvardas Danila, Dominique Costagliola, Raimonda Matulionyte

**Affiliations:** 1 Clinic of Infectious Diseases and Dermatovenerology, Institute of Clinical Medicine, Faculty of Medicine, Vilnius University, Vilnius, Lithuania; 2 Centre of Infectious Diseases, Vilnius University Hospital Santaros Klinikos, Vilnius, Lithuania; 3 State Information System of Tuberculosis, Public Health Department, Ministry of Health, Vilnius University Hospital Santaros Klinikos, Vilnius, Lithuania; 4 AIDS Centre, Department of Infectious Diseases, University Hospital of Klaipeda, Klaipeda, Lithuania; 5 Tuberculosis Department, Republican Klaipeda Hospital, Klaipeda, Lithuania; 6 Adult Infectious Diseases Unit, Clinic of Conservative Medicine, Republican Siauliai County Hospital, Siauliai, Lithuania; 7 Department of Infectious Diseases, Lithuanian University of Health Sciences, Kaunas, Lithuania; 8 Kaunas Hospital of the Lithuanian University of Health Sciences, Kaunas, Lithuania; 9 National Public Health Surveillance Laboratory, Vilnius, Lithuania; 10 Faculty of Medicine, Vilnius University, Vilnius, Lithuania; 11 Clinic of Chest Diseases, Immunology, and Allergology, Faculty of Medicine, Vilnius University, Vilnius, Lithuania; 12 Centre of Pulmonology and Allergology, Vilnius University Hospital Santaros Klinikos, Vilnius, Lithuania; 13 Sorbonne Université, INSERM, Institut Pierre Louis d’épidémiologie de Santé Publique, Paris, France; Chung Shan Medical University, TAIWAN

## Abstract

**Background:**

Tuberculosis (TB) is a public health problem in Lithuania, among the 18 high-priority TB countries in the European region, and the most common AIDS-indicative disease with the highest proportion in the EU/EEA since 2015. The study aimed to identify socio-demographic, clinical characteristics and their relationship with TB outcomes in TB-HIV co-infected patients in Lithuania.

**Methods:**

A retrospective chart review analysed the characteristics of TB-HIV co-infected adults registered in State Information System of Tuberculosis over 2008–2020. The factors associated with drug-resistant TB and unsuccessful treatment outcome were identified by multivariable logistic regression.

**Results:**

The study included 345 cases in 311 patients (239 new, 106 previously treated cases), median age 40 years (IQR 35–45), 80.7% male. 67.8% patients knew their HIV-positive status before TB diagnosis, median time to TB diagnosis was 8 years (IQR 4–12). 83.6% were unemployed, 50.5%—anytime intravenous drug users (IDU), 34.9% abused alcohol. Drug-resistant TB rates in new and previously treated TB cases were 38.1% and 61.3%, respectively. In multivariable analysis, higher risk of drug-resistant TB was associated with imprisonment in new (aOR 3.35; 95%CI 1.17–9.57) and previously treated (aOR 6.63; 95%CI 1.09–40.35) cases. In 52.3% of new TB cases and in 42.5% previously treated TB cases the treatment outcomes were unsuccessful. In multivariable analysis of new TB cases, current imprisonment (aOR 2.77; 95%CI 1.29–5.91) and drug-resistant TB (aOR 2.18; 95%CI 1.11–4.28) were associated with unsuccessful treatment outcome. In multivariable analysis of previously treated TB cases, female gender (aOR 11.93; 95%CI 1.86–76.69), alcohol abuse (aOR 3.17; 95%CI 1.05–9.58), drug-resistant TB (aOR 4.83; 95%CI 1.53–15.28) were associated with unsuccessful treatment outcome.

**Conclusions:**

In the TB-HIV-infected adult cohort in Lithuania, unemployment, imprisonment, IDU, alcohol abuse, known to be risk factors for TB, were very frequent. Drug resistance was an undeniable risk factor for unsuccessful treatment outcome and imprisonment was associated with drug resistant TB.

## Introduction

Tuberculosis (TB) remains a public health issue and the leading cause of death among people living with HIV (PLHIV) in the world. In 2019, there were 10 million new cases of TB and 1.2 million TB deaths globally. Among them, 8.2% of new TB cases and 208,000 deaths were in PLHIV [1]. Besides, for the first time over a decade, TB death rate has increased in 2020 because of decreased access to prevention and care due to COVID-19 pandemic [2]. Drug-resistant TB persists as a serious public health threat accounting for nearly half million cases in 2019 worldwide and 78% of these cases were multidrug-resistant TB (MDR-TB). Treatment of MDR-TB is a challenge and requires the attention to the complexity of pill burden, drug-drug interactions and cost [1, 3, 4]. In 2019, 3.3% of new cases and 17.7% of recurrent TB cases were MDR-TB, and the highest proportions (>50% in recurrent TB cases) were in the countries of the former Soviet Union [1].

The global TB treatment success rate for new TB and drug-resistant TB cases in 2018 was 85% and 57%, respectively [1]. Among PLHIV the overall reported TB treatment success rates were generally low and for MDR-TB rarely exceeded 50% [4, 5].

Lithuania belongs to the 18 high-priority TB countries in the European region and has one of the highest rates of TB. The rate of TB notification in 2019 was 37.7 per 100,000, four-times higher than the rate of TB notification in the EU/EEA. Treatment outcomes in Lithuania were worse than expected, with treatment success rate of 87.1% in all TB patients and 70.4% in MDR-TB patients in 2019 [6]. TB is the most common AIDS-indicative disease in Lithuania, with the highest proportion in the EU/EEA since 2015 and was still >60% in 2019 [7]. It is important to know the differences of TB among PLHIV and the factors associated with the infection by multidrug-resistant *Mycobacterium tuberculosis* and unsuccessful treatment in this population as it will help TB program coordinators and scientists involved in this field to focus on strategic issues and the improvement of TB control program. Therefore, the aim of our study was to identify socio-demographic, clinical characteristics and their relationship with TB outcomes in TB-HIV co-infected patients in Lithuania.

## Methods

### Study design and population

A retrospective chart review was conducted of all TB-HIV co-infected adults aged 18 years or older from January 1, 2008, through December 31, 2020, registered in the State Information System of Tuberculosis. The State Information System of Tuberculosis was established in 2002 to register all TB cases in Lithuania, including new and re-treatment cases, monitor and control management of TB, to evaluate the epidemiological indicators and to forecast the epidemiological situation of TB in Lithuania. The State Information System of Tuberculosis is a unified, centralized and standardized tuberculosis cases notification and registration database. The database contains the following information about a TB patient: socio-demographic, diagnostic data, drug susceptibility data of *M*. *tuberculosis*, treatment and outcomes.

### Ethical statement

The study was approved by the Regional Biomedical Research Ethics Committee of Vilnius University (2020-06-22, Nr.2020/6˗1240˗723). The Regional Biomedical Research Ethics Committee of Vilnius University waived the requirement for the informed consent on the basis of the right granted of the Law on Ethics of Biomedical Research of Lithuania. The information obtained was made anonymous and deidentified prior to analysis to ensure confidentiality.

### Operational definitions

TB cases, drug resistance and treatment outcomes were defined according to the WHO Definitions and reporting framework for tuberculosis [8].

The following definitions were used for a standard clinical case:

- bacteriologically confirmed TB case–a case with a positive smear microscopy, culture or WHO approved rapid diagnostics (i.e. Xpert MTB/RIF);
- clinically diagnosed TB case–a case diagnosed on the basis of radiological, suggestive histological findings without laboratory confirmation.
- These standard clinical cases were classified according to:
- anatomical site of disease
  - pulmonary TB–any bacteriologically or clinically confirmed TB case in which the lung parenchyma or the tracheobronchial tree is involved; *miliary TB*–a form of TB resulting of *M*. *tuberculosis* hematogenous dissemination from the lungs to other organs and it was assigned to pulmonary TB;
  - extrapulmonary TB—any bacteriologically or clinically confirmed TB case in which other organs than the lungs are involved;
- history of previous treatment
  - new patients–have never been treated for TB or have taken TB treatment for less than 1 month;
  - previously treated patients–have been treated for TB for 1 month or more in the past; they are classified as follows:
    - relapse patients–have been treated for TB previously and were declared as cured or treatment completed and now are diagnosed with a recurrent episode of TB (either a true relapse or a new episode of TB caused by reinfection);
    - treatment after failure patients–have been treated for TB and their treatment failed at the end of the most recent treatment course;
    - treatment after loss to follow-up–have been treated for TB and were defined as loss to follow-up at the end of the most recent treatment course;
    - other previously treated patients–have been treated for TB although their treatment outcome is unknown or undocumented;
    - patients with unknown previous TB treatment history–do not fill the criteria of above-mentioned categories.

According to the drug resistance, cases were classified:

- drug-susceptible TB*–*no evidence of *M*. *tuberculosis* resistance to rifampicin (not drug-resistant or multidrug-resistant TB) and no need for the treatment with a second-line drug regimen;
- drug-resistant TB–*M*. *tuberculosis* resistant at least to rifampicin;
- multidrug-resistant TB (MDR-TB)–*M*. *tuberculosis* resistant to not less than both isoniazid and rifampicin;
- extensively drug-resistant TB (XDR-TB)–*M*. *tuberculosis* resistant to any fluoroquinolone and to at the minimum one of three second-line injectable drugs.

According to the current WHO guidelines [9], management of MDR-TB should be the same as rifampicin resistant TB. Therefore, factors associated with MDR-TB were evaluated putting rifampicin resistant TB and MDR-TB in one category.

Treatment outcomes were categorized into the following:

- cured: negative smear microscopy or negative culture at the end of treatment and on at least one previous follow-up test*;*
- treatment completed: TB treatment completed without evidence of failure;
- treatment failed: positive smear microscopy or positive culture after 5 months or later during treatment;
- lost to follow-up: patient did not start treatment or interrupted their treatment for two consecutive months or more;
- died: patient died from any cause before starting or during the course of TB treatment;
- not evaluated: patient for whom no treatment outcome is ascribed; this includes patients who left the country before treatment completion;
- successful treatment outcome: the patients who were cured or completed treatment; the cases of completed treatment were evaluated as treatment success even if the results of negative smear microscopy or negative culture at the end of treatment were not received;
- unsuccessful treatment outcome: the patients with treatment failure, lost to follow-up, died or not evaluated.

Treatment outcomes were evaluated separately for new and previously treated cases.

### Data collection

A structured data collection questionnaire was prepared to extract the data from patients’ medical records. It contained sociodemographic characteristics, type of tuberculosis infection, TB registration group (new patients, previously treated patients and patients with unknown previous TB treatment history), TB clinical signs, history of exposure to anti-tuberculosis treatment, TB treatment outcome, the status of HIV infection, information of antiretroviral therapy (ART), CD4 count and HIV viral load at the time of TB diagnosis, blood sample test results at the time of TB diagnosis, co-infection with hepatitis C virus (HCV) defined by positive HCV IgG antibody (anti-HCV), and hepatitis B virus (HBV) defined by positive hepatitis B surface antigen (HBsAg). Sputum-smear microscopy, *M*. *tuberculosis* cultures, and drug susceptibility testing were performed in local laboratories. All laboratories were quality assured by the WHO Supranational Reference Laboratory Network.

### Outcome

We focused on three outcomes: infection with drug-resistant TB and unsuccessful TB treatment outcomes in new and previously treated cases.

### Statistical analysis

The collected data were coded and entered into computer using Epidata version 3.1. Analysis was done using IBM SPSS version 20.0. Frequencies, proportions and summary statistics were used to describe the study population in relation to socio-demographic and clinical characteristics. Categorical variables were analyzed using Pearson chi-square and Fisher exact tests when appropriate. Continuous variables were expressed as median and interquartile range (IQR). Nonparametric Mann-Whitney U test was used to identify differences between groups in continuous outcomes. Univariable logistic analysis was used to explore unadjusted association between variables (socio-demographic characteristics, imprisonment, smoking, any-time intravenous drug use, alcohol abuse, HIV acquisition way, time of HIV diagnosis, hepatitis status, sputum-smear microscopy, TB clinical signs, CD4 count and HIV viral load at the time of TB diagnosis, blood sample test results at the time of TB diagnosis) and outcome. Variables with a *p* value less than 0.05 in univariable analysis were included in multivariable analysis restricted to individuals with complete data.

## Results

### Socio-demographic and clinical characteristics of study participants

**Fig 1:**
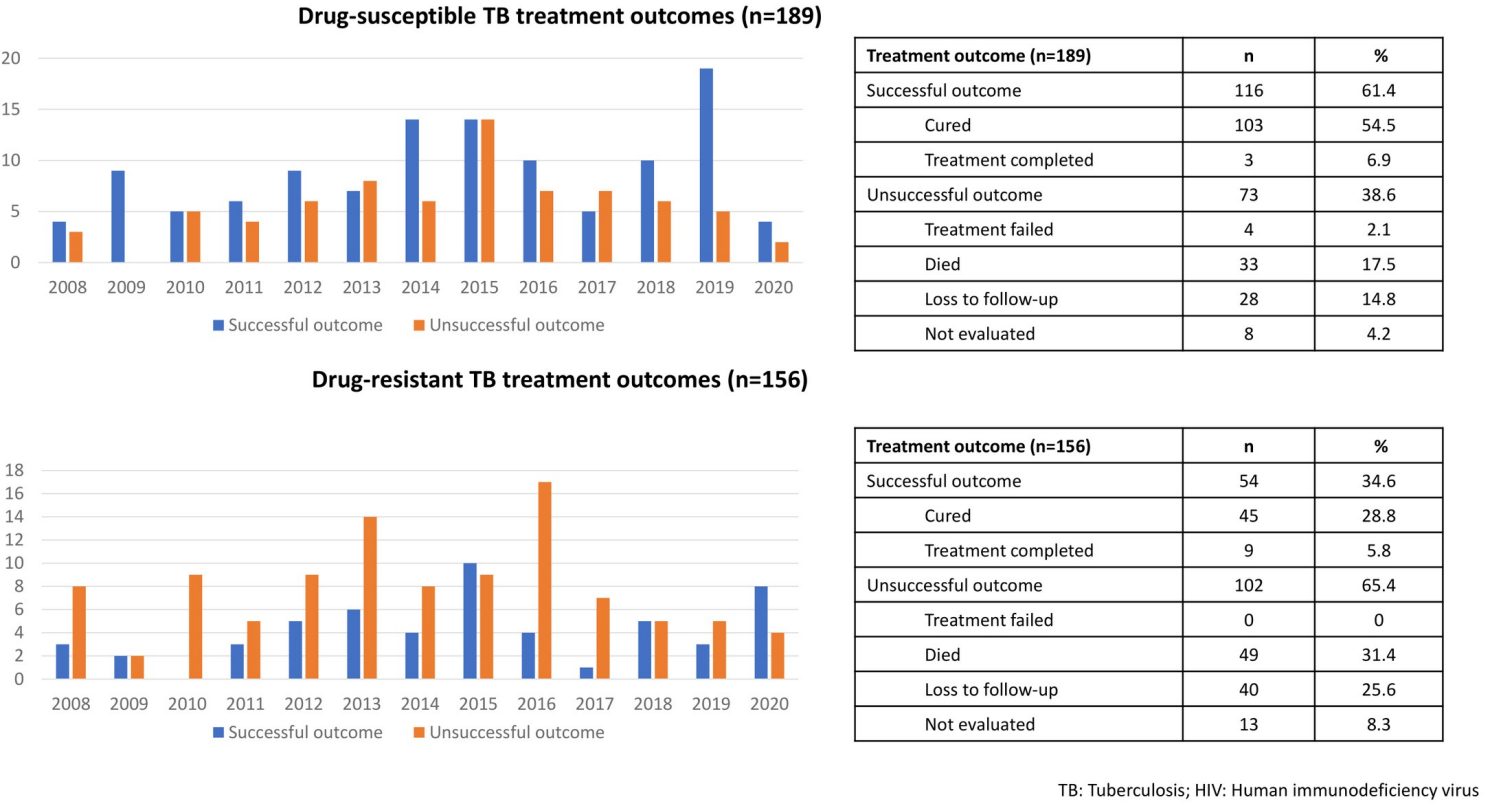
Dynamics of TB treatment outcomes in TB/HIV cases (n = 345) in Lithuania.

Out of the cumulative number of 3,431 PLHIV in Lithuania at the end of 2020 [10], 345 cases with active TB in 311 patients (9,1%) were registered in the State Information System of Tuberculosis over the period 2008 to 2020. All the cases were included in the study (Fig 1). All patients were white, 251 (80.7%) were male and the median age was 40 years (IQR 35–45). The sociodemographic and clinical characteristics of study participants are shown in Tables 1 and 2.

**Table 1:** Socio-demographic characteristics of TB-HIV co-infected patients in Lithuania, 2008–2020 (n = 311).

Characteristics	n	N	%
**Sex**			
Male	251	311	80.7
Female	60	311	19.3
**Age (years)**			
18–24	5	311	1.6
25–34	71	311	22.8
35–49	187	311	60.1
≥50	48	311	15.4
**Place of residence**			
Urban	260	309	84.1
Rural	49	309	15.9
**Predisposing factors**			
Homelessness	26	300	8.7
Unemployment	254	304	83.6
Imprisonment in history	120	192	62.5
Current	52	120	43.3
Previous	68	120	56.7
Smoking	255	305	83.6
Any-time intravenous drug use	155	307	50.5
Alcohol abuse	107	307	34.9
Exposure to active TB any time	66	270	24.4
**HIV transmission route**			
Intravenous drug use	161	300	53.7
Heterosexual	117	300	39.0
Men who have sex with men	1	300	0.3
Unknown	21	300	7.0
**Time of HIV diagnosis**			
Before TB diagnosis *	181	267	67.8
On TB diagnosis	86	267	32.2
**HCV co-infection**	179	273	65.6
**HBV co-infection**	5	222	2.3
**TB registration status**			
New	239	311	76.8
Previously treated	72	311	23.2
Relapse	41	72	56.9
Treatment failure	8	72	11.1
Treatment after loss to follow-up	23	72	31.9

TB: Tuberculosis; HIV: Human immunodeficiency virus; HCV: Hepatitis C virus; HBV: Hepatitis B virus;

* The median time to TB diagnosis was 8 years (IQR 4–12).

**Table 2:** Clinical characteristics of TB-HIV cases in Lithuania, 2008–2020 (n = 345).

Characteristics	New TB cases		Previously treated TB cases		p-value
	N	%	N	%	
Type of TB					
Pulmonary TB	205/239	85.8	95/106	89.6	0.15
Smear-positive	116/205	56.6	64/95	67.4	0.08
Smear-negative	89/205	43.4	31/95	32.6	0.08
Miliary TB	30/239	12.6	7/106	6.6	0.11
Extrapulmonary TB	34/239	14.2	11/106	10.4	0.36
**Diagnosis supported by**					
Culture positive*	199/239	83.3	105/106	99.1	<0.001
Smear positive	121/239	50.6	64/106	60.4	0.09
GeneXpert positive**	43/146	29.5	8/13	61.5	0.03
**Drug resistance**					
Drug-susceptible TB	148/239	61.9	41/106	38.7	<0.001
Drug-resistant TB	91/239	38.1	65/106	61.3	<0.001
MDR-TB	62/239	25.9	38/106	35.8	0.05
XDR-TB	5/239	2.1	15/106	14.2	<0.001
**Clinical signs**					
Cough	160/207	77.3	31/39	79.5	0.76
Fever	148/207	71.5	25/39	64.1	0.35
Weight loss	140/207	67.6	24/39	61.5	0.46
Night sweats	106/207	51.2	20/39	51.3	0.99
**BMI, kg/m^2^**					
<18.5	22/106	20.8	0/19	0.0	0.09
18.5–24.9	73/106	68.9	17/19	89.5	
≥25.0	11/106	10.4	2/19	10.5	
**CD4 count at the time of TB diagnosis, cells/mm^3^**					
<50	28/119	23.5	1/31	3.2	0.08
50–99	19/119	16.0	5/31	16.1	
100–349	51/119	42.9	17/31	54.8	
≥350	21/119	17.6	8/31	25.8	
**HIV-RNA at the time of TB diagnosis, copies/mL**					
<200	13/85	15.3	5/17	29.4	0.09
200–999	5/85	5.9	1/17	5.9	
1,000–9,999	9/85	10.6	5/17	29.4	
10,000–99,999	16/85	18.8	1/17	5.9	
≥100,000	42/85	49.4	5/17	29.4	
**Baseline Hb, g/l**					
<100	36/134	26.9	1/26	3.8	0.01
≥100	98/134	73.1	25/26	96.2	0.01
**Baseline PLT, x10e9/l**					
<150	33/131	25.2	5/26	19.2	0.52
≥150	98/131	74.8	21/26	80.8	0.52
**Baseline CRP count, mg/l**					
<50	46/100	46.0	9/15	60.0	0.31
≥50	54/100	54.0	6/15	40.0	0.31
**On ART**	55/171	32.1	16/41	39.0	0.41

TB: Tuberculosis; HIV: Human immunodeficiency virus; MDR-TB: Multidrug-resistant tuberculosis; XDR-TB: Extensively drug-resistant tuberculosis; CD4: Cluster differentiation-4; Hb: Hemoglobin; PLT: Platelet; CRP: C-reactive protein; BMI: body mass index; ART: antiretroviral therapy

*Drug resistance found in 90/239 (37.7%) new and in 64/106 (60.4%) previously treated cases;

**Drug resistance found in 16/146 (10.9%) new and in 4/13 (30.8%) previously treated cases.

The majority (83.6%) of participants were unemployed and 62.5% had a history of imprisonment. Smoking, any-time intravenous drug usage (IDU) and abuse of alcohol were identified in 83.6%, 50.5% and 34.9% patients, respectively. Sixty-eight percent of patients knew their HIV-positive status before TB diagnosis, the median time to TB diagnosis was 8 years (IQR 4–12). Regarding the gender related characteristics, imprisonment in history (71.5% vs. 20.6%, p<0.001), IDU (54.0% vs. 35.6%, p = 0.01), HIV acquisition by IDU (56.8% vs. 40.7%, p = 0.03) and HCV co-infection (68.5% vs. 53.7%, p = 0.04) were more common among men (S1 Table).

According to the history of previous TB treatment, 239 (69.3%) were new cases and 106 (30.7%) were previously treated cases: 64 (61%) were relapsed, 10 (9.5%) were failed and 32 (30.2%) were returnees after loss to follow-up. Regarding clinical characteristics, drug-susceptible TB (61.9% vs. 38.7%, p<0.001) and hemoglobin (Hb) < 100 g/l at the time of TB diagnosis (26.9% vs. 3.8%, p = 0.01) were more common in new TB cases, and positive culture (83.3% vs. 99.1, p<0.001) and GeneXpert (29.5% vs. 61.5%, p = 0.03)–in previously treated TB cases (Table 2).

Regarding the HIV related characteristics of new TB cases, median CD4 count at TB diagnosis was 136 (IQR 53–289) cells/mm^3^, median HIV-RNA– 94,700 (IQR 1,845–230,000) copies/ml and 15.3% of patients had undetectable (<200 copies/ml) viral load. Thirty-two percent of new cases had started antiretroviral therapy (ART) before TB treatment, median time of ART to TB diagnosis was 201 days (IQR 47–677). In previously treated TB cases, median CD4 count at TB diagnosis was 197 (IQR 105–344) cells/mm^3^, median HIV-RNA– 155,000 (IQR 16,925–254,000) copies/ml and 33.3% of patients had undetectable viral load. Thirty-nine percent of previously treated TB cases received ART before TB diagnosis, median time of ART to TB diagnosis was 770 days (IQR 161–1,814).

Out of 239 patients with new TB, 28 developed a recurrent TB episode. Among 72 previously treated patients, 6 patients had one more recurrence during the study. Overall, the number of recurrence per patient was 0.34. In all study population, the median time to the first TB recurrence (n = 100) was 54 months (IQR 35–95) and to the second recurrence (n = 6)– 74 months (31.5–166). The unadjusted odds ratio for TB recurrence (n = 311) were significantly higher for unemployed (OR 4.2; 95% CI 1.46–12.12; p = 0.008), smokers (OR (4.18; 95% CI 1.45–12.05; p = 0.008) and patients with drug-resistant TB (OR 2.28; 95% CI 1.33–3.89; p = 0.003). Multivariable analysis (n = 298) confirmed the same risk factors: unemployment (aOR 4.11; 95% CI 1.40–12.03; p = 0.01), smoking (aOR 3.69; 95% CI 1.26–10.83; p = 0.02) and drug-resistant TB (aOR 2.13; 95%CI 1.22–3.73; p = 0.01).

Overall, the treatment success was 116 (61.4%) for drug-susceptible TB and 54 (34.6%)–for drug-resistant TB cases (Fig 1).

### Factors associated with drug-resistant TB

Both univariable and multivariable analysis were done to identify the association between drug-resistant TB and the socio-demographic and clinical characteristics of new and previously treated TB cases.

In univariable analysis of new cases (n = 237), imprisonment (OR 3.28; 95% CI 1.44–7.46; p = 0.01), IDU (OR 1.89; 95% CI 1.07–3.36; p = 0.03) and HIV acquisition by IDU (OR 2.31; 95% CI 1.27–4.18; p = 0.01) were associated with drug-resistant TB.

Univariable analysis of previously treated cases (n = 104) revealed imprisonment (OR 3.50; 95% CI 1.09–11.29; p = 0.04), IDU (OR 2.99; 95% CI 1.35–6.65; p = 0.01) and loss to follow-up (OR 3.67; 95% 1.39–9.64; p = 0.008) association with drug-resistant TB.

**Fig 2:**
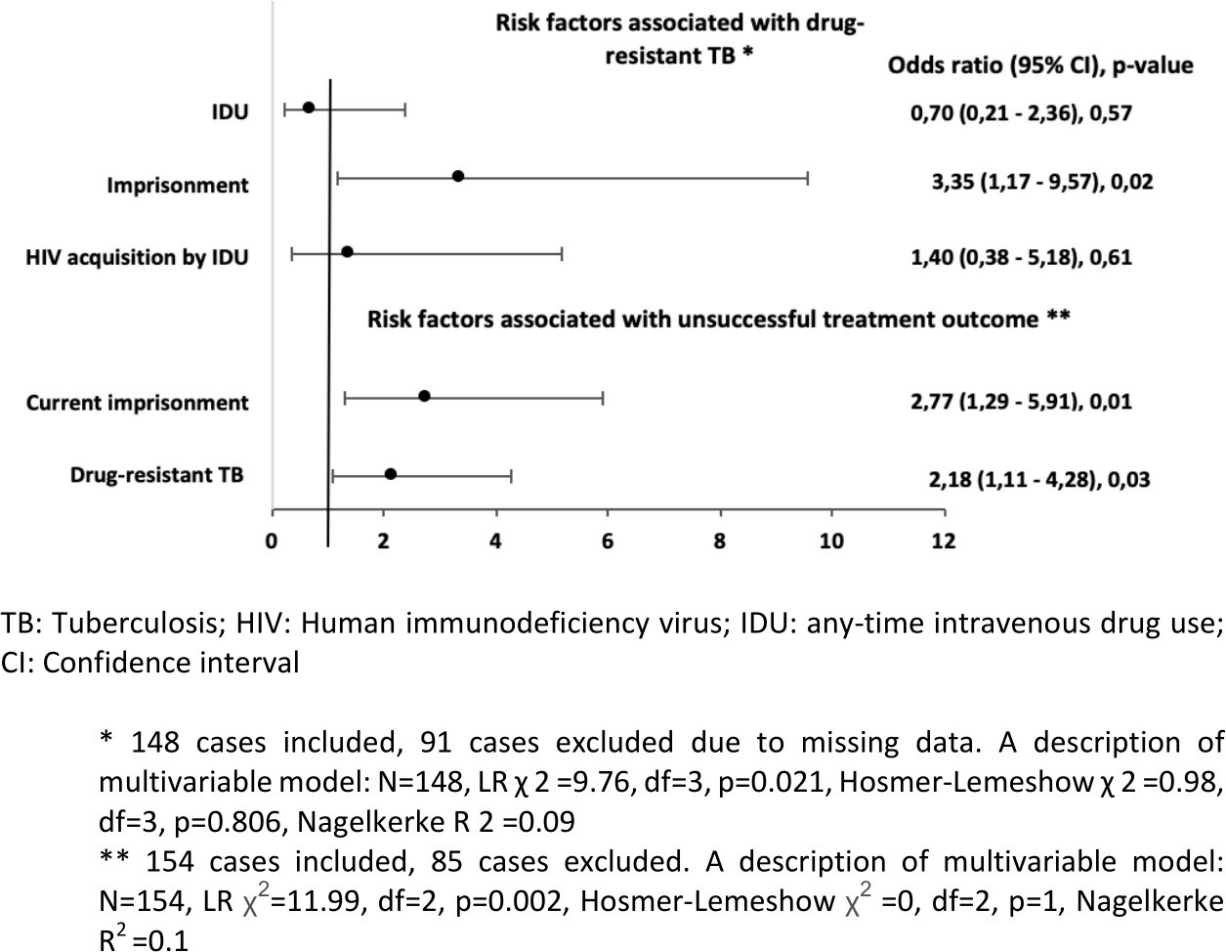
Multivariable analysis of factors associated with drug-resistant TB and unsuccessful treatment outcomes in new TB-HIV cases in Lithuania, 2008–2020 (n = 239).

**Fig 3:**
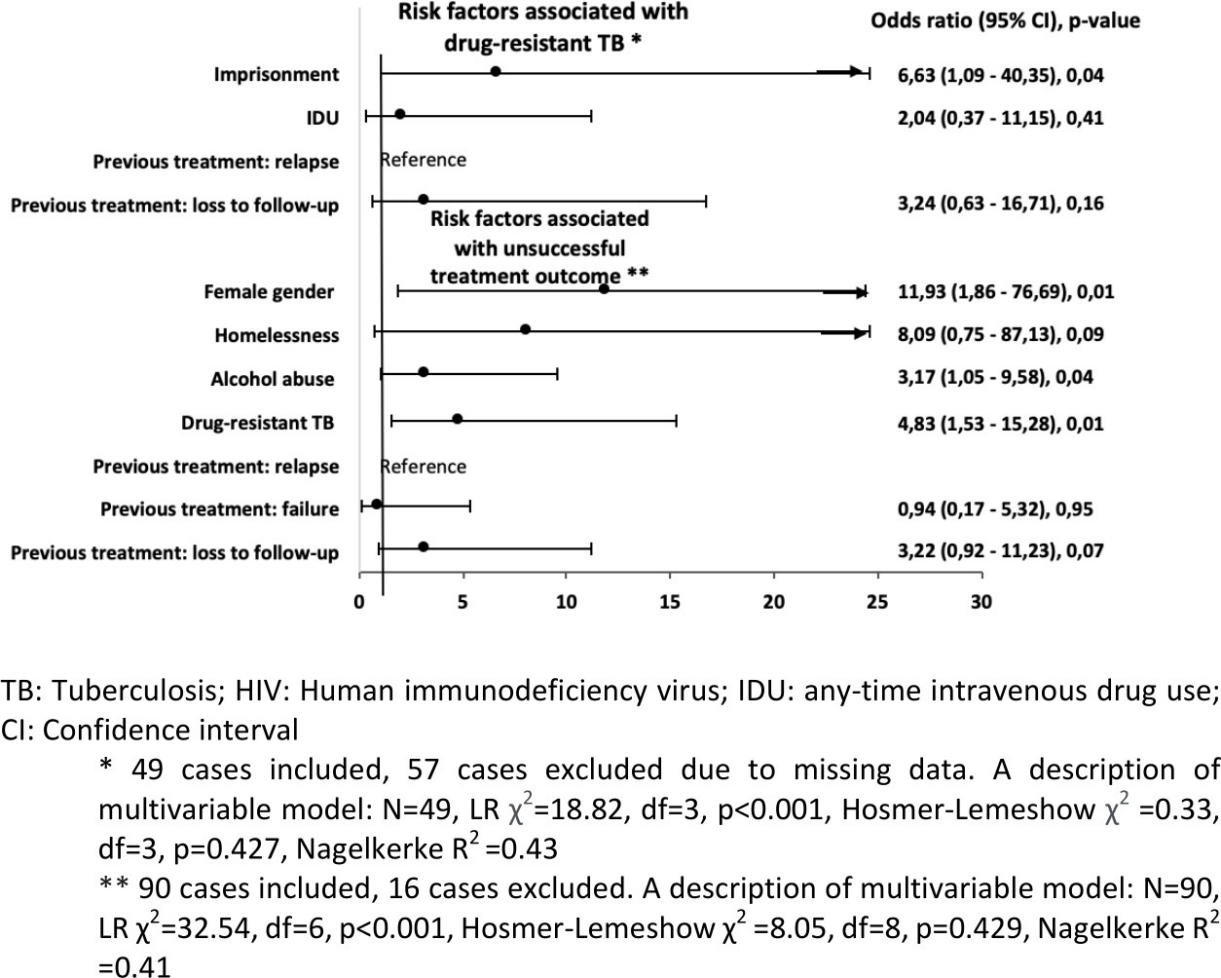
Multivariable analysis of factors associated with drug-resistant TB and unsuccessful treatment outcomes in previously treated TB-HIV cases in Lithuania, 2008–2020 (n = 106).

Figs 2 and 3 show the final multivariable model: imprisonment was the only factor associated with drug resistant TB in new and previously treated TB cases.

### Factors associated with unsuccessful TB treatment outcome

In 125/239 (52.3%) of new TB cases and in 45/106 (42.5%) of previously treated TB cases, the treatment outcomes were unsuccessful.

In univariable analysis of new TB cases, imprisonment in history (OR 2.59; 95% CI 1.3–5.19; p = 0.01) and drug-resistant TB were associated with unsuccessful treatment (OR 2.49; 95% CI 1.45–4.24; p = 0.001). Current imprisonment was a risk factor to unsuccessful treatment outcome (OR 2.65; 95%CI 1.26–5.57; p = 0.01), while previous imprisonment was not found to be associated with unsuccessful treatment outcome (OR 1.38; 95% CI 1.08 = 1.73; p = 0.21). Multivariable analysis revealed that current imprisonment (aOR 2.77; 95% CI 1.29–5.91, p = 0.01) and drug-resistant TB (aOR 2.18, 95% CI 1.11–4.28; p = 0.03) were risk factors to unsuccessful treatment outcome (Fig 2).

In univariable analysis of previously treated TB cases, female gender (OR 5.27; 95%CI 1.12–24.86; p = 0.04), homelessness (OR 8.41; 95% CI 1.02–69.21; p = 0.048), alcohol abuse (OR 2.57; 95% CI 1.12–5.91; p = 0.03), drug-resistant TB (OR 4.2; 95% CI 1.83–9.63; p = 0.001) and loss to follow-up (OR 6.20; 95% CI 2.07–18.54; p = 0.001) were associated with a higher risk of unsuccessful treatment; and in multivariable analysis–female gender (aOR 11.93; 95% CI 1.86–76.69; p = 0.01), alcohol abuse (aOR 3.17; 95% CI 1.05–9.58; p = 0.04) and drug-resistant TB (aOR 4.83; 95% CI 1.53–15.28; p = 0.01) (Fig 3).

### Mortality rates and associated risk factors

During the study period, 79 (25.4%) out of 311 patients died: 62/239 (25.9%) new patients and 17/72 (23.6%) previously treated patients.

Socio-demographic and clinical characteristics, including the number of TB episodes, and their association with death assessed through univariable and multivariable analysis. The unadjusted OR of death (n = 311) were significantly higher for alcohol abusers (OR 2.11; 95% CI 1.25–3.58; p = 0.005), patients with Hb < 100 g/l at the time of TB diagnosis (OR 2.73; 95% CI 1.23–6.07; p = 0.014) and drug-resistant TB (OR 2.02; 95% CI 1.2–3.39; p = 0.008). Multivariable analysis (n = 149) confirmed the same risk factors: drug-resistant TB (aOR 2.70; 95% CI 1.19–6.10; p = 0.02), alcohol abuse (aOR 2.36; 95%CI 1.06–5.23; p = 0.04) and Hb < 100 g/l (aOR 2.81; 95% CI 1.17–6.75; p = 0.02).

## Discussion

This study aimed to determine socio-demographic, clinical characteristics and their relationship with TB outcomes in TB-HIV co-infected patients in Lithuania. We found that the treatment success rate among cases with drug-susceptible and drug-resistant TB (61.4% and 34.6%, respectively) was low. Thirty-eight percent of new cases and 61% of previously treated cases were drug-resistant TB, which is consistent with other studies conducted in Eastern European region [11–15] but higher compared with other European regions [16]. The rate of previously treated cases for TB was high (30.7%) and the prevalence of relapse after treatment completion among them was also high. According to WHO 2020 Global Tuberculosis report, the global TB treatment success rate for PLHIV was 76% and in the WHO Europe region it was 51% [1]. In the End TB Strategy, WHO aims TB treatment success rate to be ≥90% of all notified cases by 2025 [17]. Therefore, our findings emphasize the importance of improving TB treatment and control programs in Lithuania in order to achieve WHO the End TB Strategy goals.

Unemployment, imprisonment, alcohol abuse and intravenous drug use, known social risk factors for TB, were frequent in our study population [1, 18–23]. Several studies found that unemployment rate increased more than twice during the TB episode [24, 25]. Imprisonment is commonly the primary risk factor for TB or an additional factor for TB evolution in the population with other high-risk factors, i.e., substance abuse and HIV infection. A recent study carried out in Brazil showed that imprisonment was the primary risk factor, as TB risk increased markedly during imprisonment period and among ex-prisoners, and declined in the years after the release [26]. Furthermore, the review of factors defining transmission of HIV, HCV and TB in Eastern Europe and Central Asia found that 75% of incident TB cases among intravenous drug users were associated with imprisonment [27–29]. We found that new patients with TB and having a history of imprisonment had 2.59 times higher odds of having unsuccessful TB treatment outcome and current imprisonment was associated with 2.77 higher odds of unsuccessful treatment outcome. Additionally, the adjusted odds of having drug-resistant TB were 3.35 and 6.63 higher for new and previously treated TB patients with a history of imprisonment, respectively. During the study period, imprisoned patients in Lithuania were treated using centralized purchase funded by the Ministry of Justice and not the Ministry of Health, which possibly resulted in the delay in TB treatment. In addition, the adherence of these patients and the continuation of TB treatment after imprisonment remain a challenge in Lithuania due to social (unstable place of residence, lack of relatives and social workers who ensure the care for the patient), as well as financial issues (cost of travel to the clinic) and a lack of awareness of the importance of TB treatment. Also, drug-resistant TB, anemia and alcohol abuse were risk factors of death. Therefore, our study findings emphasize the need of increased focus on optimizing social conditions among un-employers, providing support to overcome alcohol and intravenous drug use and focus on involving family or other support persons in TB treatment. It also highlights the necessity to improve treatment strategies in imprisonment and the importance of social help during and after imprisonment period.

At the time of first TB episode, our study patients were with marked immunodeficiency and high HIV-RNA viral load: CD4 count <350 cells/mm^3^ was in 42.9% and <50 cells/mm^3^ –in 23.5% of patients and only 15.3% had viral load < 200 copies/mL. These findings are characteristic of eastern Europe, which were clearly distinguished in a large prospective cohort study of TB co-infected PLHIV across eastern and western Europe, and Latin America and a low CD4 cell count was found as a factor related to the increased TB-related mortality: the adjusted odds of TB-related death in the cases with CD4 count ≤50 cells/µl were 2.45 and 3.46 in all study participants and patients from Eastern Europe region, respectively [16, 27]. More than half (67.8%) of patients knew their HIV diagnosis prior to TB diagnosis and only 32.2% were on ART at the time of TB diagnosis. These findings are largely in agreement with the studies published from the Eastern European region [18, 30, 31]. The low uptake of ART could be associated with the governmental regulations of ART initiation in Lithuania as the “treat all” approach was only adopted in February 2018 [32]. The screening for latent TB with interferon-gamma release assay (IGRA) has not been included into Lithuanian HIV management guidelines until mid-2018. Also, no further steps after IGRA positivity detection and treatment of latent TB among PLHIV have been foreseen. Therefore, the high percentage of patients who knew their HIV status before TB diagnosis emphasizes the need to increase awareness of TB in this population and the need of policy for programmatic management of latent TB in PLHIV.

The emerging of recurrent TB posed a huge challenge for TB elimination programs worldwide. In our study, the number of TB recurrence per patient was 0.34 and it was similar to recurrent TB rates reported in other studies performed in different geographical regions [33–36]. We found smoking, unemployment and drug-resistant TB as risk factors for recurrent TB. A recent large multicenter prospective study in China showed that patients who continued smoking during TB treatment were at higher risk for TB recurrence and this risk remained significant even in those who stopped smoking [37]. Also, the WHO/Union Monograph on TB and Tobacco highlights smoking as one of the most important risk factors that increases the probability of TB recurrence [38]. Previous studies have also established that socio-economic determinants–low income, unemployment–were factors increasing the risk of TB recurrence [39, 40]. In accordance with other studies [33–36], drug-resistant TB was associated with TB recurrence and it emphasizes the importance of search for more effective TB management strategies. Also, the long period of time to TB recurrence (54 months to the first recurrence) in our study highlights the need of longer follow-up observation after the treatment completion. On contrary to other studies [41–43], the recurrent TB was not associated with death.

In our study, female gender, drug-resistant TB and alcohol abuse were associated with unsuccessful treatment outcome in previously treated TB cases. In other studies [5, 28, 44–53], women were more likely to develop severe forms of TB and recurrent TB, and the duration of TB treatment was longer. However, factors related to unsuccessful treatment, such as imprisonment and IDU, except alcohol abuse, were more common among men in our study. These differences can possibly be explained by behavioral (men tend to have a higher number of social contacts and engage in high-risk behaviors) and biological hypotheses (immune responses modulated by sex hormones and increased genetic susceptibility to TB among men) [54, 55]. Our study findings suggest the importance of search for new strategies of TB management in the settings with high TB prevalence, which would cover access to care facilitation, the new TB drug regimens, abuse treatment options and integrated services. It highlights the necessity of screening for active TB. Also, integrated services of substance abuse treatment for TB-HIV co-infected patients should be considered.

In our study the prevalence of extrapulmonary TB was similar (14.2% in new TB cases and 10.4% in previously treated TB cases) compared to the global notification rate of WHO Europe region– 16% [1]. The rate of miliary TB was 12.6% in new TB cases and 6.6% in previously treated TB cases and this form of TB was not associated with the poorer treatment outcome. In other large multicenter prospective studies [16, 56], the rate of miliary TB was higher and the association with the increased risk of death was found. These differences could be explained by a larger number of participants and different stratifications used for analysis.

The strength of this study is that it largely describes the characteristics of all nationally registered TB cases of HIV co-infected patients in the context of the high prevalence of TB and drug-resistant TB over the period of more than ten years. In addition, the study highlights the important factors and features that have to be improved in the management of TB cases while strengthening the TB control program. Furthermore, TB treatment outcomes and associated risk factors were evaluated separately for new and previously treated TB cases.

Our study has also several limitations. First, the retrospective nature of the study limited the data on detailed medical information on the participants. A number of participants had missing CD4 cell counts, HIV-RNA, blood sample test and biochemistry measurements hindering a more accurate evaluation of laboratory results association with the infection by multidrug-resistant *M*. *tuberculosis* and unsuccessful treatment outcome. Second, due to retrospective type of our study and different treatment environments of included prisons, our study was not designed to assess antituberculosis treatment related factors, such as treatment regimen, duration and adherence, which undoubtedly had an impact on outcomes. Third, we did not analyze the trends in drug susceptibility testing because of limited availability of such data in the database. Finally, the information about the prescription of opioid substitution therapy was not available and we could not evaluate the impact of opioid substitution therapy to TB outcome.

## Conclusions

In the TB-HIV-infected adult cohort in Lithuania, unemployment, imprisonment, IDU, alcohol abuse, known to be risk factors for TB, were very frequent. Drug resistance was an undeniable risk factor for unsuccessful treatment outcome and imprisonment was associated with drug resistant TB. Further interventions to address these challenges should focus on improving TB management, especially in socially marginalised groups including prison, and providing integrated person-centred rather than disease-centred care.

The assessment of TB treatment outcomes, analysis and prevention of factors associated with unsuccessful treatment results is of crucial importance in reaching the success in TB control programme. In the frame of WHO End TB Strategy [56], this study identified risk groups requiring integrated programmatic case management.

## Supporting information

S1 Table Socio-demographic and clinical characteristics of TB-HIV co-infected patients according to gender in Lithuania, 2008–2020 (n = 311). (DOCX)

S1 Dataset (XLS)

## References

[1] World Health Organization. Global tuberculosis report 2020.

[2] World Health Organization. Global tuberculosis report 2021.

[3] World Health Organization. Guidelines for the programmatic management of drug-resistant tuberculosis 2011 update. 23844450

[4] Lange C, Abubakar I, Alffenaar JW, Bothamley G, Caminero JA, Carvalho AC et al; TBNET. Management of patients with multidrug-resistant/extensively drug-resistant tuberculosis in Europe: a TBNET consensus statement. Eur Respir J. 2014; 44(1):23–63. 24659544 10.1183/09031936.00188313 PMC4076529

[5] Ahuja SD, Ashkin D, Avendano M, Banerjee R, Bauer M, Bayona JN et al; Collaborative Group for Meta-Analysis of Individual Patient Data in MDR-TB. Multidrug resistant pulmonary tuberculosis treatment regimens and patient outcomes: an individual patient data meta-analysis of 9,153 patients. PloS Med. 2012;9(8):e1001300. 22952439 10.1371/journal.pmed.1001300 PMC3429397

[6] The State Information System of Tuberculosis. Tuberculosis treatment outcomes– 2019 data.

[7] European Centre for Disease Prevention and Control/WHO Regional Office for Europe. HIV/AIDS surveillance in Europe 2020–2019 data. Stockholm: ECDC; 2020.

[8] World Health Organization. Definitions and reporting framework for tuberculosis– 2013 revision (updated December 2014 and January 2020).

[9] WHO consolidated guidelines on tuberculosis. Module 4: treatment—drug-resistant tuberculosis treatment, 2022 update. Geneva: World Health Organization; 2022. Licence: CC BY-NC-SA 3.0 IGO. 36630546

[10] The Centre for Communicable Diseases and AIDS– 2021 data.

[11] Efsen AMW, Schultze A, Miller RF, Panteleev A, Skrahin A, Podlekareva DN, et al; TB: HIV study in EuroCoord. Management of MDR-TB in HIV co-infected patients in Eastern Europe: Results from the TB:HIV study. J Infect. 2018;76(1):44–54. doi: 10.1016/j.jinf.2017.10.007 29061336 PMC6293190

[12] Podlekareva DN, Folkvardsen DB, Skrahina A, Vassilenko A, Skrahin A, Hurevich H, et al. Tuberculosis Drug Susceptibility, Treatment, and Outcomes for Belarusian HIV-Positive Patients with Tuberculosis: Results from a National and International Laboratory. Tuberc Res Treat. 2021;2021:6646239. doi: 10.1155/2021/6646239 33868727 PMC8035031

[13] Skrahina A, Hurevich H, Zalutskaya A, Sahalchyk E, Astrauko A, van Gemert W, et al. Alarming levels of drug-resistant tuberculosis in Belarus: results of a survey in Minsk. Eur Respir J 2012;39(6): 1425–31. doi: 10.1183/09031936.00145411 22005924 PMC3393766

[14] Zignol M, Dean AS, Alikhanova N, Andres S, Cabibbe AM, Cirillo DM, et al. Population-based resistance of Mycobacterium tuber-culosis isolates to pyrazinamide and fluoroquinolones: results from a multicountry surveillance project. Lancet Infect Dis 2016; 16(10):1185–92. doi: 10.1016/S1473-3099(16)30190-6 27397590 PMC5030278

[15] Kraef C, Bentzon A, Skrahina A, Mocroft A, Peters L, Lundgren JD, et al. Improving healthcare for patients with HIV, tuberculosis and hepatitis C in eastern Europe: a review of current challenges and important next steps. HIV Med. 2022;23(1):48–59. doi: 10.1111/hiv.13163. Epub 2021 Sep 1. PMID: . 34468073

[16] Podlekareva DN, Efsen AM, Schultze A, Post FA, Skrahina AM, Panteleev A et al; TB:HIV study group in EuroCoord. Tuberculosis-related mortality in people living with HIV in Europe and Latin America: an international cohort study. Lancet HIV. 2016; ;3(3):e120–31. doi: 10.1016/S2352-3018(15)00252-0. Epub 2016 Feb 2. PMID: . 26939735

[17] World Health Organization. The End TB strategy. 2015.

[18] Efsen AM, Schultze A, Post FA, Panteleev A, Furrer H, Miller RF, et al; TB:HIV study group in EuroCoord. Major Challenges in Clinical Management of TB/HIV Coinfected Patients in Eastern Europe Compared with Western Europe and Latin America. PloS One. 2015 Dec 30;10(12):e0145380. doi: 10.1371/journal.pone.0145380 ; PMCID: PMC4696866. 26716686 PMC4696866

[19] Andrews JR, Shah NS, Weissman D, Moll AP, Friedland G, Gandhi NR. Predictors of multidrug- and extensively drug-resistant tuberculosis in a high HIV prevalence community. PloS one. 2010;5(12):e15735 Epub 2011/01/07. doi: 10.1371/journal.pone.0015735 21209951 PMC3012092

[20] Dalton T, Cegielski P, Akksilp S, Asencios L, Campos Caoili J, et al. Prevalence of and risk factors for resistance to second-line drugs in people with multidrug-resistant tuberculosis in eight countries: a prospective cohort study. Lancet. 2012;380(9851):1406–17. Epub 2012/09/04. doi: 10.1016/S0140-6736(12)60734-X 22938757 PMC11019390

[21] Faustini A, Hall AJ, Perucci CA. Risk factors for multidrug resistant tuberculosis in Europe: a systematic review. Thorax. 2006;61(2):158–63. Epub 2005/10/29. doi: 10.1136/thx.2005.045963 16254056 PMC2104570

[22] Pradipta IS, Forsman LD, Bruchfeld J, Hak E, Alffenaar JW. Risk factors of multidrug-resistant tuberculosis: A global systematic review and meta-analysis. J Infect. 2018 Dec;77(6):469–478. doi: 10.1016/j.jinf.2018.10.004. Epub 2018 Oct 16. PMID: . 30339803

[23] Navya N, Jeyashree K, Madhukeshwar AK, Anand T, Nirgude AS, Nayarmoole BM, et al. Are they there yet? Linkage of patients with tuberculosis to services for tobacco cessation and alcohol abuse–a mixed methods study from Karnataka, India. BMC Health Serv Res. 2019 Feb 1;19(1):90. doi: 10.1186/s12913-019-3913-8 30709351 PMC6359801

[24] Chittamany P, Yamanaka T, Suthepmany S, Sorsavanh T, Siphanthong P, Sebert J, et al. First national tuberculosis patient cost survey in Lao People’s Democratic Republic: Assessment of the financial burden faced by TB-affected households and the comparisons by drug-resistance and HIV status. PloS One. 2020; 12;15(11):e0241862. doi: 10.1371/journal.pone.0241862 33180777 PMC7660466

[25] Nhung NV, Hoa NB, Anh NT, Anh LTN, Siroka A, Lonnroth K, et al. Measuring catastrophic costs due to tuberculosis in Viet Nam. Int J Tuberc Lung Dis. 2018;22(9):983–90. Epub 2018/08/11. doi: 10.5588/ijtld.17.0859 30092862

[26] Mabud TS, de Lourdes Delgado Alves M, Ko AI, Basu S, Walter KS, Cohen T, et al. Evaluating strategies for control of tuberculosis in prisons and prevention of spillover into communities: An observational and modeling study from Brazil. PloS Med. 2019;16(1):e1002737. doi: 10.1371/journal.pmed.1002737. Erratum in: Med PloS. 2019;16(3):e1002764. 30677013 PMC6345418

[27] Altice FL, Azbel L, Stone J, Brooks-Pollock E, Smyrnov P, Dvoriak S, et al. The perfect storm: incarceration and the high-risk environment perpetuating transmission of HIV, hepatitis C virus, and tuberculosis in Eastern Europe and Central Asia. Lancet. 2016; 17;388(10050):1228–48. doi: 10.1016/S0140-6736(16)30856-X. Epub 2016 Jul 14. PMID: ; PMCID: PMC5087988. 27427455 PMC5087988

[28] Edge CL, King EJ, Dolan K, McKee M. Prisoners co-infected with tuberculosis and HIV: a systematic review. J Int AIDS Soc. 2016;19(1):20960. Published 2016 Nov 15. doi: 10.7448/IAS.19.1.20960 27852420 PMC5112354

[29] Dara M, Acosta CD, Melchers NV, Al-Darraji HA, Chorgoliani D, Reyes H et al. Tuberculosis control in prisons: current situation and research gaps. Int J Infect Dis. 2015 Mar;32:111–7. doi: 10.1016/j.ijid.2014.12.029 . 25809766

[30] Bentzon AK, Panteleev A, Mitsura V, Borodulina E, Skrahina A, Denisova E et al.; TB:HIV Study Group. Healthcare delivery for HIV-positive people with tuberculosis in Europe. HIV Med. 2021 Apr;22(4):283–293. doi: 10.1111/hiv.13016. Epub 2020 Nov 20. PMID: . 33215809 PMC9801686

[31] Mathers BM, Degenhardt L, Ali H, Wiessing L, Hickman M, Mattick RP, et al. HIV prevention, treatment, and care services for people who inject drugs: a systematic review of global, regional, and national coverage. Lancet. 2010 Mar 20;375(9719):1014–28. doi: 10.1016/S0140-6736(10)60232-2. Epub 2010 Feb 26. PMID: . 20189638

[32] Ministry of Health of the Republic of Lithuania. Roadmap to scale up HIV responce. Jan 2019.

[33] Cox HS, Morrow M, Deutschmann PW. Long term efficacy of DOTS regimens for tuberculosis: systematic review. BMJ. 2008;336:484–487. doi: 10.1136/bmj.39463.640787.BE 18250104 PMC2258398

[34] Kim L, Moonan PK, Yelk Woodruff RS, Kammerer JS, Haddad MB. Epidemiology of recurrent tuberculosis in the United States, 1993–2010. Int J Tuberc Lung Dis. 2013;17:357–360. doi: 10.5588/ijtld.12.0640 23321472

[35] Shao Y, Song H, Li G, Li Y, Li Y, Zhu L, et al. Relapse or Re-Infection, the Situation of Recurrent Tuberculosis in Eastern China. Front Cell Infect Microbiol. 2021 Mar 17;11:638990. doi: 10.3389/fcimb.2021.638990 ; PMCID: PMC8010194. 33816342 PMC8010194

[36] Korhonen V, Soini H, Vasankari T, Ollgren J, Smit PW, Ruutu P. Recurrent tuberculosis in Finland 1995–2013: a clinical and epidemiological cohort study. BMC Infect Dis. 2017 Nov 16;17(1):721. doi: 10.1186/s12879-017-2818-6 ; PMCID: PMC5693478. 29145819 PMC5693478

[37] Lin H, Lin Y, Xiao L, Chen Y, Zeng X, Chang C. How Do Smoking Status and Smoking Cessation Efforts Affect TB Recurrence After Successful Completion of Anti-TB Treatment? A Multicenter, Prospective Cohort Study With a 7-Year Follow-up in China. Nicotine Tob Res. 2021 Nov 5;23(12):1995–2002. doi: 10.1093/ntr/ntab117 . 34059890

[38] WHO/UNION. Association between exposure to tobacco smoke and tuberculosis: a qualitative systematic review, 2007. Available: http://www.who.int/tobacco/resources/ publications/tb_tobac_monograph.pdf

[39] Duarte R, Lönnroth K, Carvalho C, Lima F, Carvalho ACC, Muñoz-Torrico M, et al. Tuberculosis, social determinants and co-morbidities (including HIV). Pulmonology. 2018 Mar-Apr;24(2):115–119. doi: 10.1016/j.rppnen.2017.11.003. Epub 2017 Dec 21. PMID: . 29275968

[40] Youn HM, Shin MK, Jeong D, Kim HJ, Choi H, Kang YA. Risk factors associated with tuberculosis recurrence in South Korea determined using a nationwide cohort study. PLoS One. 2022 Jun 16;17(6):e0268290. doi: 10.1371/journal.pone.0268290 ; PMCID: PMC9202932. 35709199 PMC9202932

[41] Crofts JP, Andrews NJ, Barker RD, Delpech V, Abubakar I. Risk factors for recurrent tuberculosis in England and Wales, 1998–2005. Thorax. 2010 Apr;65(4):310–4. doi: 10.1136/thx.2009.124677 . 20388755

[42] Hung CL, Chien JY, Ou CY. Associated factors for tuberculosis recurrence in Taiwan: a nationwide nested case-control study from 1998 to 2010. PLoS One. 2015 May 1;10(5):e0124822. doi: 10.1371/journal.pone.0124822 ; PMCID: PMC4416773. 25932917 PMC4416773

[43] Du J, Zhang L, Ma Y, Chen XY, Ge QP, Tian XZ, et al. Treatment and recurrence on re-treatment tuberculosis patients: a randomized clinical trial and 7-year perspective cohort study in China. Eur J Clin Microbiol Infect Dis. 2020 Jan;39(1):93–101. doi: 10.1007/s10096-019-03696-8. Epub 2019 Dec 5. PMID: . 31807989

[44] Fekadu G, Turi E, Kasu T, Bekele F, Chelkeba L, Tolossa T et al. Impact of HIV status and predictors of successful treatment outcomes among tuberculosis patients: A six-year retrospective cohort study. Ann Med Surg (Lond). 2020 Nov 15;60:531–541. doi: 10.1016/j.amsu.2020.11.032 ; PMCID: PMC7704363. 33299558 PMC7704363

[45] Mutembo S, Mutanga JN, Musokotwane K, Kanene C, Dobbin K, Yao X et al. Urban-rural disparities in treatment outcomes among recurrent TB cases in Southern Province, Zambia. BMC Infect Dis. 2019 Dec 30;19(1):1087. doi: 10.1186/s12879-019-4709-5 ; PMCID: PMC6938018. 31888518 PMC6938018

[46] Prado TN, Rajan JV, Miranda AE, Dias ED, Cosme LB, Possuelo LG et al. Clinical and epidemiological characteristics associated with 28nfavourable tuberculosis treatment outcomes in TB-HIV co-infected patients in Brazil: a hierarchical polytomous analysis. Braz J Infect Dis. 2017 Mar-Apr;21(2):162–170. doi: 10.1016/j.bjid.2016.11.006. Epub 2016 Dec 6. PMID: . 27936379 PMC9427597

[47] Tola A, Mishore KM, Ayele Y, Mekuria AN, Legese N. Treatment Outcome of Tuberculosis and Associated Factors among TB-HIV Co-Infected Patients at Public Hospitals of Harar Town, Eastern Ethiopia. A five-year retrospective study. BMC Public Health. 2019;19(1):1658. Published 2019 Dec 10. doi: 10.1186/s12889-019-7980-x 31822286 PMC6902430

[48] Collaborative Group for the Meta-Analysis of Individual Patient Data in MDR-TB treatment–2017, Ahmad N, Ahuja SD, et al. Treatment correlates of successful outcomes in pulmonary multidrug-resistant tuberculosis: an individual patient data meta-analysis. Lancet. 2018;392(10150):821–834. Doi:10.1016/S0140-6736(18)31644-1 10.1016/S0140-6736(18)31644-1 PMC6463280 30215381

[49] Ali SA, Mavundla TR, Fantu R, Awoke T. Outcomes of TB treatment in HIV co-infected TB patients in Ethiopia: a cross-sectional analytic study. BMC Infect Dis. 2016;16(1):640. doi: 10.1186/s12879-016-1967-3 ; PMCID: PMC5097375. 27814693 PMC5097375

[50] Engelbrecht MC, Kigozi NG, Chikobvu P, Botha S, van Rensburg HCJ. Unsuccessful TB treatment outcomes with a focus on HIV co-infected cases: a cross-sectional retrospective record review in a high-burdened province of South Africa. BMC Health Serv Res. 2017;17(1):470. doi: 10.1186/s12913-017-2406-x ; PMCID: PMC5504727. 28693508 PMC5504727

[51] Rehm J, Baliunas D, Borges GL, Graham K, Irving H, Kehoe T, et al. The Relation Between Different Dimensions of Alcohol Consumption and Burden of Disease-An Overview. *Addiction* (Abingdon England) 2010;105(5):817–43. doi: 10.1111/j.1360-0443.2010.02899.x 20331573 PMC3306013

[52] Lönnroth K, Williams BG, Stadlin S, Jaramillo E, Dye C. Alcohol use as a risk factor for tuberculosis–a systematic review. BMC Public Health. 2008; 8:289. doi: 10.1186/1471-2458-8-289 18702821 PMC2533327

[53] Rehm J, Samokhvalov AV, Neuman MG, Room R, Parry C, Lönnroth K, et al. The association between alcohol use, alcohol use disorders and tuberculosis (TB). A systematic review. BMC Public Health. 2009; 9:450–450. doi: 10.1186/1471-2458-9-450 19961618 PMC2796667

[54] Maheen Humayun, Joconiah Chirenda, Wen Ye, Innocent Mukeredzi, Hilda Angela Mujuru, Zhenhua Yang, Effect of Gender on Clinical Presentation of Tuberculosis (TB) and Age-Specific Risk of TB, and TB-Human Immunodeficiency Virus Coinfection, Open Forum Infectious Diseases, Volume 9, Issue 10, October 2022, ofac512, 10.1093/ofid/ofac512 PMC9620549 36324321

[55] Nhamoyebonde S, Leslie A. Biological differences between the sexes and susceptibility to tuberculosis. J Infect Dis. 2014 Jul 15;209 Suppl 3:S100-6. doi: 10.1093/infdis/jiu147 . 24966189

[56] Kraef C, Bentzon A, Panteleev A, Skrahina A, Bolokadze N, Tetradov S, et al; T. B.:H. I. V. Study Group. Delayed diagnosis of tuberculosis in persons living with HIV in Eastern Europe: associated factors and effect on mortality-a multicentre prospective cohort study. BMC Infect Dis. 2021;6;21(1):1038. doi: 10.1186/s12879-021-06745-w ; PMCID: PMC8496077. 34615474 PMC8496077

