# Supplementary material for: The socio-demographic, clinical characteristics and outcomes of tuberculosis among HIV infected adults in Lithuania: A thirteen-year analysis
Source: PLoS One. 2023 Mar 23;18(3):e0282046. doi: 10.1371/journal.pone.0282046 (PMC10035857; doi:10.1371/journal.pone.0282046)
Supplement: S1 Table — (DOCX) [file pone.0282046.s001.docx]

**S1 Table. Socio-demographic and clinical characteristics of TB-HIV co-infected patients according to gender in Lithuania, 2008-2020 (n=311)**

| **Variable** | **Variable categories** | **Gender** | | **p-value** |
| --- | --- | --- | --- | --- |
|  |  | **Female (%)** | **Male (%)** |  |
| Age, years – median (IQR) |  | 39.5 (34-47.75) | 40 (35-45) | 0.96 |
| Place of residence (n=309) | Urban | 50 (84.7) | 210 (84.0) | 0.88 |
|  | Rural | 9 (15.3) | 40 (16.0) |  |
| Homelessness (n=300) |  | 3 (5.2) | 23 (9.5) | 0.29 |
| Unemployment (n=304) |  | 49 (84.5) | 205 (83.3) | 0.83 |
| Imprisonment in history (n=192) |  | 7 (20.6) | 113 (71.5) | <0.001 |
| Smoking (n=305) |  | 37 (64.9) | 218 (87.9) | <0.001 |
| Any-time intravenous drug use (n=307) |  | 21 (35.6) | 134 (54.0) | 0.01 |
| Alcohol abuse (n=307) |  | 16 (27.1) | 91 (36.7) | 0.17 |
| HIV transmission route (n=300) | IDU | 24 (40.7) | 137 (56.8) | 0.03 |
|  | Other | 35 (59.3) | 104 (43.2) |  |
| Time of HIV diagnosis (n=267) | Before TB diagnosis | 35 (67.3) | 146 (67.9) | 0.93 |
|  | During TB episode | 17 (32.7) | 69 (32.1) |  |
| HCV co-infection (n=273) |  | 29 (53.7) | 150 (68.5) | 0.04 |
| HBV co-infection (n=222) |  | 1 (2.3) | 4 (2.2) | 1.00 |
| Positive smear microscopy (n=311) |  | 26 (43.3) | 138 (55.0) | 0.11 |
| Clinical signs (n=235) | Cough | 40 (80.0) | 142 (76.8) | 0.63 |
|  | Fever | 37 (74.0) | 130 (70.3) | 0.61 |
|  | Weight loss | 37 (74.0) | 121 (65.4) | 0.26 |
|  | Night sweats | 28 (56.0) | 92 (49.7) | 0.43 |
| BMI, kg/m^2^ – median (IQR) (n=125) |  | 19.88 (18.41-21.3) | 20.8 (19.5-23.2) | 0.08 |
| CD4 count at the time of TB diagnosis, cells/mm^3^(n=140) | <350 | 24 (82.8) | 89 (80.2) | 0.75 |
|  | ≥350 | 5 (17.2) | 22 (19.8) |  |
| HIV-RNA at the time of TB diagnosis, copies/ml (n=96) | <200 | 2 (11.1) | 14 (17.9) | 0.48 |
|  | ≥200 | 16 (88.9) | 64 (82.1) |  |
| Baseline Hb, g/l (n=152) | <100 | 6 (18.2) | 31 (26.1) | 0.35 |
|  | ≥100 | 27 (81.8) | 88 (73.9) |  |
| Baseline PLT, x10e9/l (n=149) | <150 | 7 (21.9) | 30 (25.6) | 0.66 |
|  | ≥150 | 25 (78.1) | 87 (74.4) |  |
| Baseline CRP count, mg/l (n=108) | <50 | 11 (47.8) | 40 (47.1) | 0.95 |
|  | ≥50 | 12 (52.2) | 45 (52.9) |  |
| Drug resistance | Drug-susceptible TB | 38 (63.3) | 140 (55.8) | 0.29 |
|  | Drug-resistant TB | 22 (36.7) | 111 (44.2) |  |
|  | MDR-RR-TB | 20 (33.3) | 84 (33.9) | 0.94 |
|  | XDR-TB | 0 (0.0) | 14 (5.6) | 0.06 |
| TB treatment failure (excluding patients treated for DR-TB) (n=178) |  | 15 (39.5) | 55 (39.3) | 0.98 |
| TB treatment failure (excluding patients treated for DS-TB) (n=133) |  | 18 (81.8) | 68 (61.3) | 0.07 |

TB: Tuberculosis; HIV: Human immune virus; HCV: Hepatitis C virus; HBV: Hepatitis B virus; NAA: Nucleic amplification acid; MDR-TB: Multidrug-resistant tuberculosis; XDR-TB: Extensively drug-resistant tuberculosis; CD4: Cluster differentiation-4; RNA: Ribonucleic acid; Hb: Hemoglobin; PLT: Platelet; CRP: C-reactive protein; DR-TB: drug-resistant tuberculosis; DS-TB – drug susceptible tuberculosis; BMI: body mass index.
